# Clinical evidence in ischemic stroke: Where we have gone so far and hopes for the future

**DOI:** 10.1111/ene.16047

**Published:** 2023-08-31

**Authors:** Tianhua Li, Chengyu Song, David S. Liebeskind, Adam A. Dmytriw, Ran Xu, Xue Wang, Jie Wang, Hengxiao Zhao, Wenbo Cao, Haozhi Gong, Chao Zhang, Xuesong Bai, Liqun Jiao

**Affiliations:** ^1^ Department of Neurosurgery, Xuanwu Hospital Capital Medical University Beijing China; ^2^ China International Neuroscience Institute (China‐INI) Beijing China; ^3^ Department of Science and Technology, Medical Library Peking University Huilongguan Clinical Medical School, Beijing Huilongguan Hospital Beijing China; ^4^ Department of Neurology and Comprehensive Stroke Center, David Geffen School of Medicine University of California Los Angeles California USA; ^5^ Neuroendovascular Program, Massachusetts General Hospital & Brigham and Women's Hospital Harvard Medical School Boston Massachusetts USA; ^6^ Medical Library, Xuanwu Hospital Capital Medical University Beijing China; ^7^ Escope Innovation Academy Beijing China; ^8^ Department of Interventional Neuroradiology, Xuanwu Hospital Capital Medical University Beijing China

**Keywords:** guideline, hotspot, ischemic stroke, randomized clinical trials

## Abstract

**Objective:**

Ischemic stroke is a significant cause of disability and death worldwide. Randomized clinical trials (RCTs) are important in changing guidelines and treatment strategies. This study aimed to analyze the progress of RCTs in ischemic stroke and to guide future research directions.

**Methods:**

Ischemic stroke‐related RCT articles were identified in six high‐impact medical journals using the Web of Science Core Collection database. Google Scholar was used to check whether relevant articles were included in the guidelines. The characteristics of these articles were analyzed and future research hotspots were predicted.

**Results:**

389 relevant articles were included in the analysis. The number of articles increased rapidly from 1972 to 2022, from 5 (1.3%; 1972–1982) to 208 (53.5%; 2013–2022) articles. 338 (86.9%) articles were included in relevant guidelines. According to corresponding author location, Europe was the source of the highest number of publications (183; 47.0%), followed by the Americas (152; 39.1%) and the Western Pacific (54; 13.9%). The number of publications steadily increased over time in the USA, England, Canada, Australia, Germany, and France, and surged in China and Spain, especially in the last 5 years. In recent years, endovascular therapy has accounted for the majority of ischemic stroke‐related RCT articles.

**Conclusions:**

Numerous RCTs related to ischemic stroke have been conducted in recent decades, and both the number of articles and their contribution to guideline updates are increasing. Also, a shift in research topics was observed. However, great regional imbalances in this research exist, calling for more research to be conducted in specific regions to promote the generalizability of trial conclusions.

## Graphical abstract

Screening and analysis of RCT articles related to ischemic stroke.

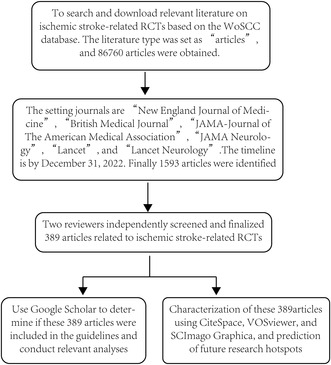

## INTRODUCTION

Stroke remains the second leading cause of death worldwide. It has been documented that 6.2 million people died from stroke worldwide in 2015, accounting for nearly 12% of all deaths worldwide [1, 2]. Ischemic strokes account for 87% of all strokes and impart significant global burden [3]. In past decades, progress in treatment strategies and techniques prompted the rewriting of clinical treatment norms and guidelines, improving the overall prognosis of stroke patients.

Randomized controlled trials (RCTs) are the direct source of high‐quality clinical evidence, providing the most reliable reference sources for guidelines. In recent decades, RCTs have been widely conducted in the field of ischemic stroke to solve several critical issues. Examples include the efficacy of surgery in treating patients with severe stenosis of intracranial or extracranial arteries [4, 5, 6], the efficacy of mechanical thrombectomy in treating patients with acute large vessel occlusion [7], and the efficacy of different medications in patients with ischemic stroke [8, 9]. These trials have influenced current clinical practice and are milestones in the field of ischemic stroke. Tracking these developments will help clinical scholars better understand the evolution of the field and guide the direction of future clinical trials. However, there is still a lack of quantitative analysis of these contents.

Based on articles published in the *New England Journal of Medicine, British Medical Journal, JAMA ‐ Journal of the American Medical Association, JAMA Neurology, Lancet*, and *Lancet Neurology,* this study attempted to analyze the characteristics of ischemic stroke‐related RCTs in recent decades and to predict hotspots for future research, with the aim of providing a comprehensive reference for future clinical trials.

## METHODS

### Identification of relevant articles for ischemic stroke‐related RCTs

A total of 86,760 articles were obtained through the Web of Science Core Collection by combining the search term “RCT” with the search term “ischemic stroke” and setting the literature type to “article”. Then, the journals set to the six highly influential journals in the field of ischemic stroke, including *New England Journal of Medicine, British Medical Journal, JAMA ‐ Journal of the American Medical Association, JAMA Neurology, Lancet,* and *Lancet Neurology*, and the cut‐off date was set to December 31, 2022. The above steps yielded a total of 1593 articles (search and download conducted on February 3, 2023). Finally, two authors (JW and HZ) independently screened the articles and identified 389 articles (including subanalysis articles from the same RCT) with ischemic stroke as the core topic for subsequent analysis. Details of the process are in Table S1.

### Clarification of whether the relevant article is cited in the guidelines

First, the Google Scholar database was searched for each of the 389 articles identified. Then, the titles were searched for their presence in publications of the following types: “guideline” or “guidelines” or “consensus” or “consensuses” or “criterion” or “criteria”. If the article was not cited in a clinical guideline or similar it was excluded.

### CiteSpace

CiteSpace is an information visualization software product developed on the basis of the theory of citation analysis, which has attracted attention for its burstness analysis [10]. The term “burstness” describes a sudden increase in keyword usage frequency or many citations over time within a certain field. A dramatic increase in the quantity of papers produced by authors, institutions, and nations/regions also fits this description [11]. The utilization of burstness analysis facilitates the expeditious comprehension of key publications, the evolution of knowledge structures, focal areas of research, and frontiers in RCTs related to ischemic stroke. A total of 389 previously obtained articles were imported into CiteSpace with the time slice = 1 year (the data are extracted in units of 1 year) [12]. Subsequently, burstness analyses of institutions and authors were conducted.

### VOSviewer

VOSviewer was developed by Leiden University and can be used to create, explore, and visualize literature data [13]. The 389 previously obtained articles were imported into VOSviewer, and the default parameters were set. Referring to previous studies, author and institutional collaboration network analysis, keyword hotspot evolution analysis, and visualization of the results were performed using this software [14, 15, 16].

### Scimago Graphica

Scimago Graphica is a visualization tool with high expressiveness and ease of use. It is not only suitable for visual data communication, but also for exploratory data analysis [17]. It is used to generate geographic visualization maps of the number of articles published in a country or region, as well as collaborative relationships.

### Standard protocol approvals, registrations, and patient consents

Ethical review and informed consent were not required by the Ethics Committee of Xuanwu Hospital, Capital Medical University, as this was a review of a publicly available database and its associated articles in which aggregated data could not be associated with any individuals.

## RESULTS

By means of the described screening process, a total of 389 articles reporting the results of ischemic stroke‐related RCTs were identified for final analysis, over the time period 1972–2022.

### Temporal and geographic distribution

**FIGURE 1:**
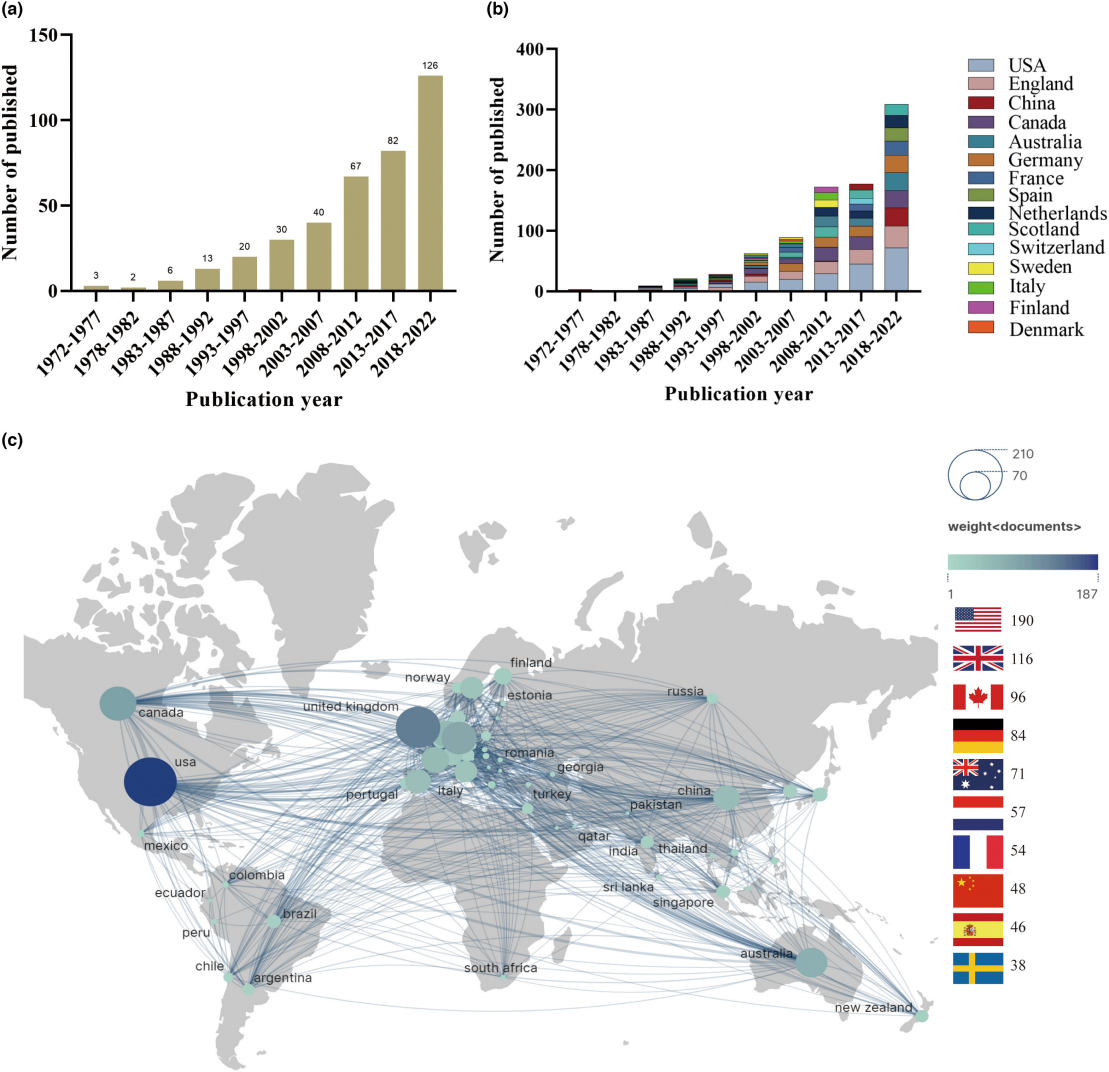
(a) Number of publications, (b) publications by country, and (c) National Collaborative Network (analyzed and visualized by VOSviewer and Scimago Graphica).

In terms of time distribution, the number of published ischemic stroke‐related RCTs has been increasing over time. The number of articles was 5 (1.3%; 1970s), 19 (4.9%; 1980s), 50 (12.9%; 1990s), 107 (27.5%; 2000s), and 208 (53.5%; 2010s) over each decade from 1972 to 2022 (Table 1). A similar phenomenon was found when using every 5‐year time interval (Figure 1a).

**TABLE 1:** Characteristics of included articles.

Characteristic	Articles (*N* = 389) n (%)
Publication years	
1972–1982	5 (1.3)
1983–1992	19 (4.9)
1993–2002	50 (12.9)
2003–2012	107 (27.5)
2013–2022	208 (53.5)
Publication	
JAMA	47 (12.1)
JAMA Neurology	55 (14.1)
Lancet	99 (25.5)
Lancet Neurology	89 (22.9)
New England Journal of Medicine	83 (21.3)
British Medical Journal	16 (4.1)
Hemispheres of corresponding author	
Eastern hemisphere	240 (61.7)
Western hemisphere	149 (38.3)
WHO regions of corresponding author	
Western Pacific	54 (13.9)
Europe	183 (47.0)
Americas	152 (39.1)
Southeast Asia	0 (0)
Eastern Mediterranean	0 (0)
Africa	0 (0)
Inclusion of guidelines	
Inclusion	338 (86.9)
Non‐inclusion	51 (13.1)

Abbreviation: WHO, World Health Organization.

According to the WHO region where the corresponding author was located, the published articles originated from Europe (183; 47.0%), Americas (152; 39.1%), and the Western Pacific (54; 13.9%), respectively. No relevant corresponding authors were found in the regions of Southeast Asia, Eastern Mediterranean, and Africa (Table 1). A total of 52 national regions contributed to ischemic stroke‐related RCTs articles in this analysis. When ranking the top 10 national regions by the total number of articles published, countries in the Americas and Europe dominate the top 10, with only China and Australia in Asia and Oceania in the rankings (Figure 1b). In the last 5 years, the USA (72) has been involved in the largest number of publications, followed by England (36), China (30), Canada (29), Australia (29), Germany (29), France (23), Spain (22), the Netherlands (21), and Scotland (18). The number of publications from the USA, England, Canada, Australia, France, and the Netherlands have increased steadily over time, while China and Spain have surged, especially in the last 5 years.

### Cooperation networks and evolution of hotspots

**FIGURE 2:**
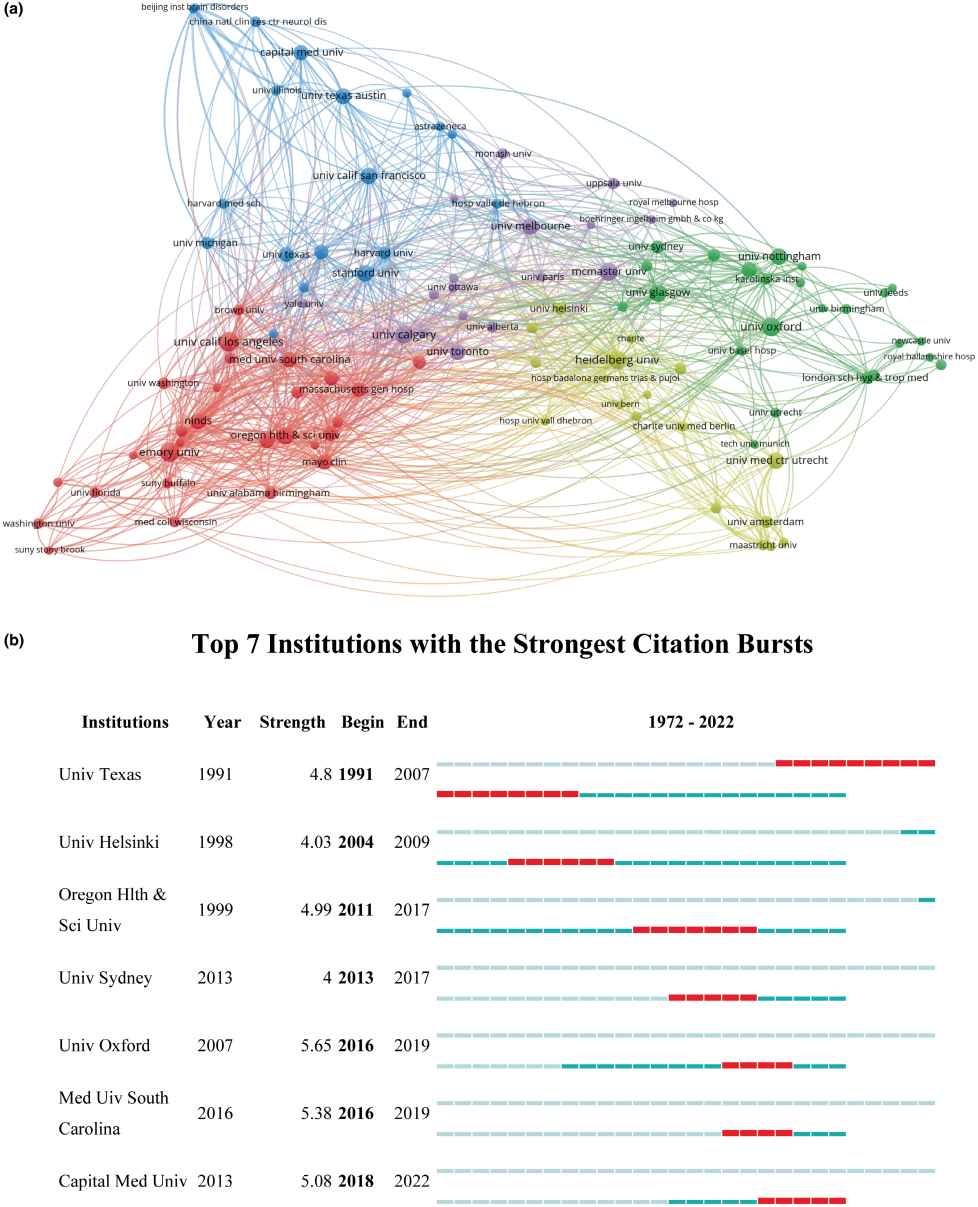
(a) Institutional collaboration networks and (b) burstness analysis (analyzed and visualized by VOSviewer and CiteSpace).

**FIGURE 3:**
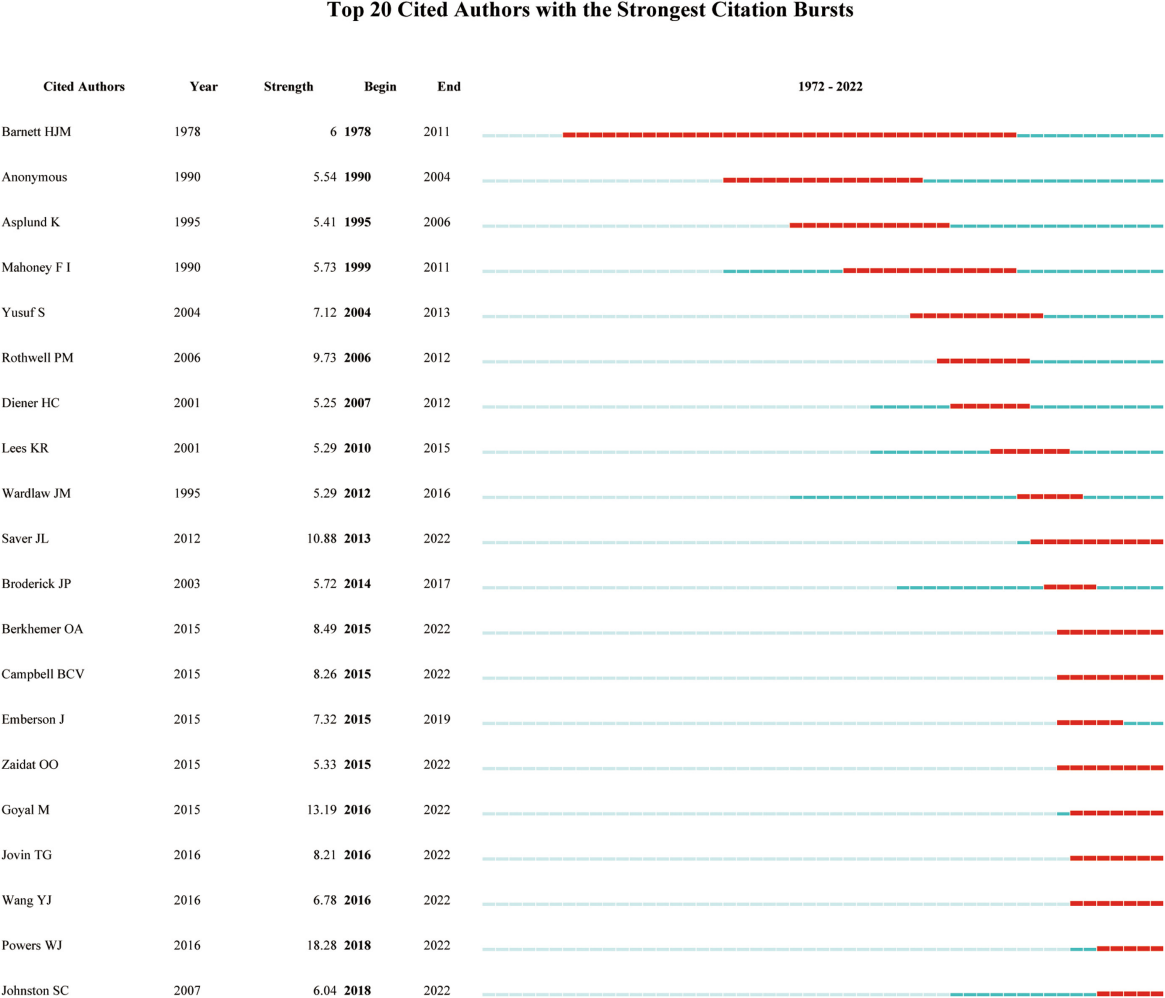
Burstness analysis of cited authors (analyzed and visualized by CiteSpace).

**FIGURE 4:**
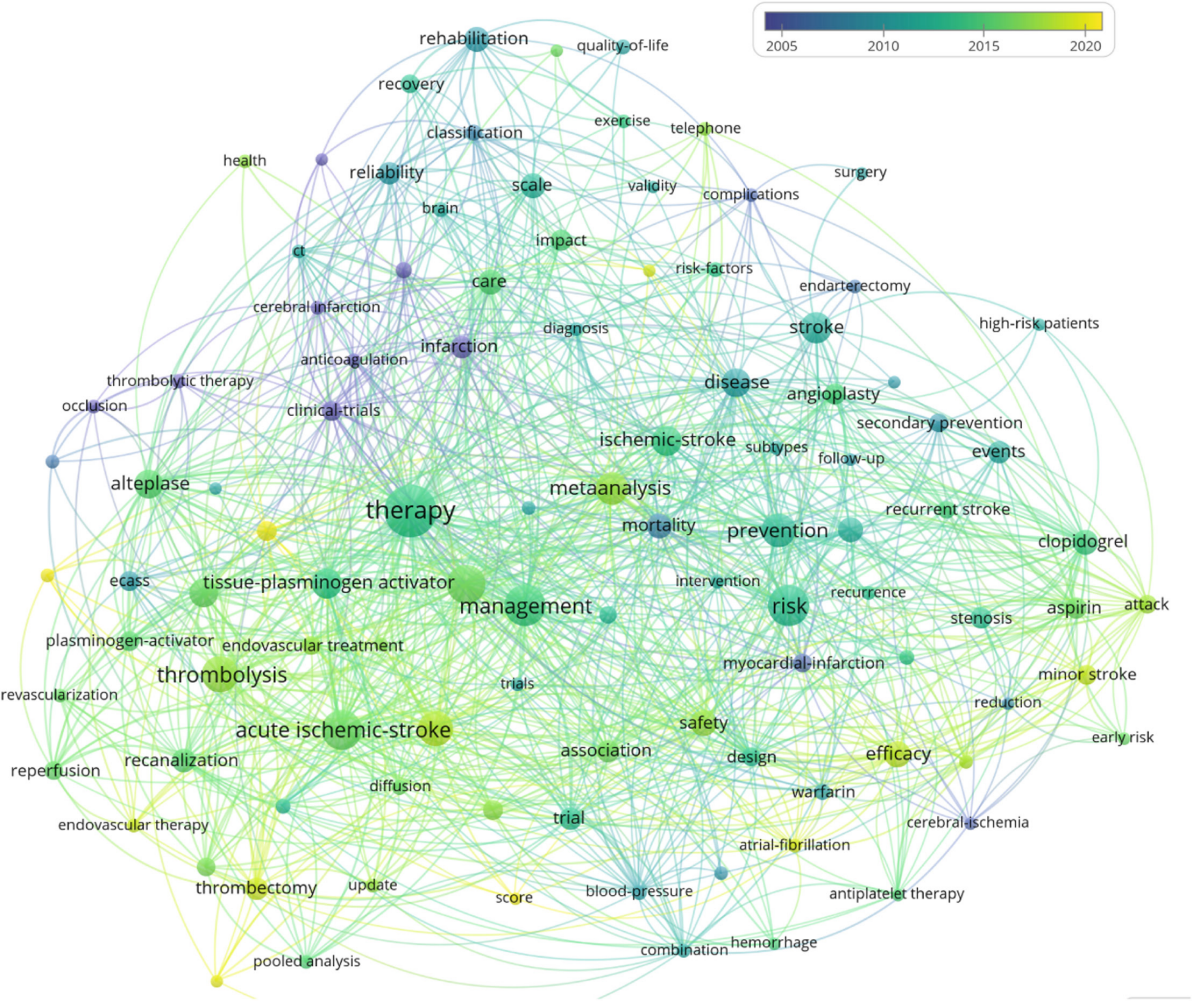
Evolution of the heat of keywords (analyzed and visualized by VOSviewer).

A denser network of collaborative relationships was found in the Americas and Europe when compared with Asia, Africa, and Oceania (Figure 1c). Figure 2a illustrates inter‐institutional collaboration, with five clusters formed based on collaborative relationships. Burstness analyses show that the earliest top cited institution was the University of Texas System, which lasted for the longest time period, from 1991 to 2007. In recent years, Capital Medical University has emerged as a prominent institution, as depicted in Figure 2b. With respect to cited authors, the leading names include Saver JL, Berkhemer OA, Campbell BCV, Zaidat OO, Goyal M, Jovin TG, Wang YJ, Powers WJ, and Johnston SC (Figure 3). Their research includes strategy selection in the areas of stroke prevention, acute ischemic stroke, intracranial artery stenosis (ICAS), and chronic occlusion (Table S2). The evolution of the popularity of keywords shows that around 2005, anticoagulation and thrombotic therapy became hot words. By around 2010, secondary protection, aspirin, and clopidogrel and topics related to these became hotspots. Around 2015, tissue plasminogen activator, alteplase, and topics related to these attracted more attention. By around 2020, endovascular treatment, thrombectomy, and topics related to these have received more attention (Figure 4).

### Contribution to guidelines

A total of 338 (86.9%) of 389 relevant articles were included in guideline publications and 51 (13.1%) were not (Table 1). The recruited articles were divided into a guideline inclusion group and a non‐inclusion group, and the baseline characteristics between the two groups were compared. On a 10‐year basis, the number of published articles included in the guidelines has been increasing: 5 (1.3%; 1970s), 19 (4.9%; 1980s), 49 (12.6%; 1990s), 107 (27.5%; 2000s), and 159 (40.9%; 2010s). There were 49 (12.6%) articles not included in guidelines in the past decade, with 41 (10.5%) articles accounting for the majority in the past 5 years. Hemispherically, the Eastern hemisphere had more articles included in guidelines (203 [52.2] vs. 135 [34.7]) compared with the Western hemisphere. According to WHO regions of corresponding author, included articles were mainly in Europe (165; 42.4%), the Americas (152; 39.1%), and the Western Pacific (40;10.3%), respectively. The average of total citations (825.4 ± 1270 vs. 51.6 ± 65.0) and average annual citations (70.0 ± 112.3 vs. 19.4 ± 24.1) in the inclusion group were higher than those in the non‐inclusion group (Table 2).

**TABLE 2:** Characteristics of articles included in the guidelines.

Characteristic	Inclusion (*n* = 338) *n* (%)	Non‐inclusion (*n* = 51) *n* (%)
Publication years		
1972–1982	5 (1.3)	0 (0)
1983–1992	19 (4.9)	1 (0.3)
1993–2002	49 (12.6)	1 (0.3)
2003–2012	107 (27.5)	0 (0)
2013–2022	159 (40.9)	49 (12.6)
Publication		
JAMA	40 (10.3)	7 (1.8)
JAMA Neurology	36 (9.3)	19 (4.9)
Lancet	91 (23.4)	8 (2.1)
Lancet Neurology	77 (19.8)	12 (3.1)
New England Journal of Medicine	80 (20.6)	3 (0.8)
British Medical Journal	14 (3.6)	2 (0.5)
Hemispheres of corresponding author		
Eastern hemisphere	203 (52.2)	37 (9.5)
Western hemisphere	135 (34.7)	14 (3.6)
WHO regions of corresponding author		
Western Pacific	40 (10.3)	19 (4.9)
Europe	165 (42.4)	18 (4.6)
Americas	133 (34.2)	14 (3.6)
Total frequency of citations	825.4 ± 1270	51.6 ± 65.0
Average annual frequency of citations	70.0 ± 112.3	19.4 ± 24.1

Abbreviation: WHO, World Health Organization.

## DISCUSSION

As the second leading cause of disability and death worldwide, stroke places a heavy burden on individuals and society [18]. Ischemic stroke is an important component of stroke, and there is still significant room for progress in effective treatment, management, and intervention methods, requiring more research and treatment resource allocation [19]. As the internationally recognized gold standard for clinical prevention and treatment research methods, RCTs play a significant role in promoting and informing guidelines. Ischemic stroke‐related RCTs are of great significance for the prevention, treatment, and rehabilitation of ischemic stroke. However, there is a lack of studies on the relevant characteristics of ischemic stroke‐related RCTs articles. Based on six highly influential journals, this study analyzed the characteristics of ischemic stroke‐related RCTs articles, predicted future research hotspots, and provided a comprehensive reference for future ischemic stroke‐related RCTs.

In general, tremendous research work has been done by various scholars in the ischemic stroke field in recent decades, promoting the fast updating of clinical guidelines. It should be noted that over half (53.5%) of the relevant RCTs were published in the most recent decade from 2013 to 2022. This may indicate that ischemic stroke has aroused increased concern from researchers worldwide due to its high rate of incidence, mortality, and morbidity. During this time, more research groups were likely able to conduct high‐quality clinical trials to drive changes in clinical guidelines. However, disparity in regions was observed, as Europe (47.0%) and the Americas (39.1%) dominated this research, followed by the Western Pacific (13.9%). It is worth acknowledging that certain countries or regions have specifically contributed significantly to the advancement of stroke research. The number of articles published in the USA, England, Canada, Australia, France, and the Netherlands has maintained steady growth, and China and Spain are growing rapidly and have entered the top 10. During the period 2018–2022, the USA, England, China, and Spain were involved in the publication of 76, 32, 30, and 22 related RCTs, respectively. In terms of WHO regions of corresponding author, Southeast Asia, the Eastern Mediterranean, and Africa are lacking in such contributions. A worldwide study of the access to mechanical thrombectomy showed that the prevalence of mechanical thrombectomy is extremely low globally, with mechanical thrombectomy access in low‐income countries being 88% lower than in high‐income countries [20]. Consequently, it would be important to say that in the future, a new focus could be on performing trials in certain geographical regions and a diversity of populations to guarantee the generalizability of clinical evidence.

Among the total of 389 articles, 338 (86.9%) were included in clinical guidelines, indicating that relevant RCTs articles contribute significantly to the progress of ischemic stroke treatment. This also indicates that these six journals have contributed significantly to ischemic stroke research by proliferating important work. Of the 51 (13.1%) articles that were not included in the guidelines, 41 (10.5%) were published in 2018–2022. This may be due to the lag of guidelines relative to article publication in time. For instance, the most recent update of the Stroke/American Heart Association (AHA) guideline was in 2019. It is worth noting that some artificial intelligence (AI)‐related RCTs on stroke patient rehabilitation have received a large number of citations within a short period of time [21, 22]. At the same time, our study found that RCTs included in guidelines were associated with a significantly higher total number of citations and average annual number of citations. It is reasonable to infer that these trials have received extensive attention from subsequent real‐world studies and provided robust evidence in support of the guidelines. Again, one drawback is that articles included in the guidelines also varied greatly as regards the WHO regions of the corresponding authors, and clinicians need to be aware of this when referring to guidelines.

It is worth noting that there is a relative abundance of international collaborations in the field of ischemic stroke research, with the USA and Europe being the most active areas of collaboration. In terms of the number of publications, only China is in the top 10 in Asia, and no relevant RCTs articles from Africa have to date been published in the six prominent journals. Stroke, the second leading cause of disability and death worldwide, imposes the greatest burden on low‐ and middle‐income countries [3, 23, 24]. Asia and Africa have the largest number of low‐ and middle‐income countries and are home to 75% of the world's population, likely warranting the focusing of resources on the conduction of relevant RCT research in these territories.

Through the burstness analysis, popular institutions and authors from different time periods can be examined. Capital Medical University was a top cited institution during the period 2018–2022, and corresponding authors Wang YJ, Ji XM, Jiao LQ, Wang BJ, and Miao ZR made important contributions [5, 25, 26, 27, 28]. A burstness analysis of the cited authors indicates that Saver JL, Berkhemer OA, Campbell BCV, Zaidat OO, Goyal M, Jovin TG, Wang YJ, Powers WJ, and Johnston SC are the most prominent figures in recent years, with noteworthy collaborative efforts observed among some of these authors. Their studies involved the treatment strategy of several aspects such as acute ischemic stroke, ICAS, and chronic artery occlusion. These topics are the subject of hot clinical debates that have led to or have the potential to change the update of clinical evidence. Their studies include optimal antiplatelet drug selection after ischemic stroke or transient ischemic attack and selection of target population (THALES, CHANCE‐2) [8, 27], bridging intravenous thrombolysis before thrombectomy (DIRECT‐MT) [29], thrombolytic drug advances (EXTEND‐IA TNK) [30] and dosage selection (EXTEND‐IA TNK Part 2) [31], effectiveness of interventional therapy for ICAS (the VISSIT trial) [32], and extracranial–intracranial bypass surgery for carotid arterial chronic occlusion (COSS) [6].

Some limitations should be highlighted. First, this study is based on six highly influential journals, and high‐quality ischemic stroke RCTs, but data from some other journals are lacking, which may result in a lack of comprehensive assessment. Second, the purpose of this study is limited to the field of ischemic stroke, with the aim of presenting more accurate information. Therefore, there is a lack of analysis of hemorrhagic stroke, which can be further deepened in the future. Third, the source of the guidelines may influence which studies are included, with European guidelines tending to include European publications, as well as the USA guidelines. Besides, the stroke‐related guidelines are inherently not global, but mainly European or American, which is a real bias, and one that the World Stroke Organization is attempting to correct.

## CONCLUSIONS

Many ischemic stroke‐related RCTs have been conducted in recent decades. The number of relevant articles and their contribution to guideline updates is increasing. Also, the study topics are changing. However, there is a significant regional imbalance among studies, and more research needs to be conducted in underrepresented areas to improve the extensibility of the preliminary conclusions.

## AUTHOR CONTRIBUTIONS

**Tianhua Li:** Conceptualization (equal); investigation (equal); resources (equal); writing – review and editing (equal). **Chengyu Song:** Investigation (equal); resources (equal). **David S. Liebeskind:** writing – review and editing (equal). **Adam A. Dmytriw:** Writing – review and editing (equal). **Ran Xu:** Writing – review and editing (equal). **Xue Wang:** Methodology (equal); data curation (equal). **Jie Wang:** Data curation (equal). **Hengxiao Zhao:** Data curation (equal). **Wenbo Cao:** Formal analysis (equal). **Haozhi Gong:** Formal analysis (equal). **Chao Zhang:** Methodology (equal). **Xuesong Bai:** Conceptualization (equal); writing – review and editing (equal). **Liqun Jiao:** Conceptualization (equal); writing – review and editing (equal); funding acquisition.

## FUNDING INFORMATION

This study was supported by Research and Translation Application of Clinical Diagnosis and Treatment Technology of the Capital (Z201100005520020; Z201100005520019) and Beijing Hospitals Authority's Ascent Plan (DFL20220702).

## CONFLICT OF INTEREST STATEMENT

The author(s) declared no potential conflicts of interest with respect to the research, authorship, and/or publication of this article.

## Supporting information


Table S1.

Table S2.

## Data Availability

Researchers can apply to use the Web of Science Core Collection database and Google Scholar database to access the data used.
