# Supplementary material for: Clinical evidence in ischemic stroke: Where we have gone so far and hopes for the future
Source: Eur J Neurol. 2023 Aug 31;31(11):e16047. doi: 10.1111/ene.16047 (PMC11464386; doi:10.1111/ene.16047)
Supplement: Supplementary file 1 — Table S1. [file ENE-31-e16047-s002.docx]

Table S1. Search strategy and results

| # | Search strategy | Search Results |
| --- | --- | --- |
| 1 | TS=(random* or RCT or RCTs or (controlled NEAR/5 (trial* or stud*)) or (clinical* NEAR/5 trial*) or ((control or treatment or experiment* or intervention) NEAR/5 (group* or subject* or patient*)) or ((singl* or doubl* or tripl* or trebl*) NEAR/5 (blind* or mask*)) or ((control or experiment* or conservative) NEAR/5 (treatment or therapy or procedure or manage*)) or placebo* or sham or assign* or allocate*) | 5640603 |
| 2 | TI=(trial or trials) | 513757 |
| 3 | #1 OR #2 | 5768239 |
| 4 | TI=(stroke* or apoplex*) OR AB=(stroke* or apoplex*) | 352979 |
| 5 | TI=((brain or encephalic or cerebr* or cerebell* or vertebrobasil* or hemispher* or intracran* or intracerebral or infratentorial or supratentorial or "middle cerebr*" or MCA$ or "anterior circulat*" or "posterior circulat*" or "basilar arter*" or "vertebral arter*" or vertebrobasilar or "space occupying" or "basal ganglia") NEAR/5 (isch$emi* or infarct* or thrombo* or emboli* or occlus* or hypoxi* or stenos* or narrow or constrit* or strict* or harden* or insufficienc* or arteriosclerosis or atherosclero* or obstruct* or block*)) OR AB=((brain or encephalic or cerebr* or cerebell* or vertebrobasil* or hemispher* or intracran* or intracerebral or infratentorial or supratentorial or "middle cerebr*" or MCA$ or "anterior circulat*" or "posterior circulat*" or "basilar arter*" or "vertebral arter*" or vertebrobasilar or "space occupying" or "basal ganglia") NEAR/5 (isch$emi* or infarct* or thrombo* or emboli* or occlus* or hypoxi* or stenos* or narrow or constrit* or strict* or harden* or insufficienc* or arteriosclerosis or atherosclero* or obstruct* or block*)) | 151349 |
| 6 | #4 OR #5 | 454560 |
| 7 | #3 AND #6 AND Literatu type is set to article AND Journal set to “New England Journal of Medicine”, “British Medical Journal”, “JAMA-Journal of The American Medical Association”, “JAMA Neurology”, “Lancet”, and “Lancet Neurology” AND Time ends on 2022-12-31 | 1593 |
| 8 | Two reviewers independently screen articles related to RCTs with a focus on ischemic stroke | 389 |
