# Supplementary material for: Clinical evidence in ischemic stroke: Where we have gone so far and hopes for the future
Source: Eur J Neurol. 2023 Aug 31;31(11):e16047. doi: 10.1111/ene.16047 (PMC11464386; doi:10.1111/ene.16047)
Supplement: Supplementary file 2 — Table S2. [file ENE-31-e16047-s001.docx]

Table S2. Related articles of recent top cited authors

| Cited author | Year | Relevant articles |
| --- | --- | --- |
| Saver JL | 2022 | Effect Of Intravenous Tirofiban Vs Placebo Before Endovascular Thrombectomy On Functional Outcomes In Large Vessel Occlusion Stroke The Rescue Bt Randomized Clinical Trial |
|  | 2022 | Thrombectomy Alone Versus Intravenous Alteplase Plus Thrombectomy In Patients With Stroke: An Open-Label Blinded-Outcome, Randomised Non-Inferiority Trial |
|  | 2021 | Prospective, Multicenter, Controlled Trial Of Mobile Stroke Units |
|  | 2020 | Thrombectomy For Stroke In The Public Health Care System Of Brazil |
|  | 2019 | An Injectable Implant To Stimulate The Sphenopalatine Ganglion For Treatment Of Acute Ischaemic Stroke Up To 24 H From Onset (Impact-24B): An International, Randomised, Double-Blind, Sham-Controlled, Pivotal Trial |
|  | 2019 | Dabigatran For Prevention Of Stroke After Embolic Stroke Of Undetermined Source |
|  | 2019 | Prehospital Transdermal Glyceryl Trinitrate In Patients With Ultra-Acute Presumed Stroke (Right-2): An Ambulance-Based, Randomised, Sham-Controlled, Blinded, Phase 3 Trial |
|  | 2018 | Frequency, Predictors, And Outcomes Of Prehospital And Early Postarrival Neurological Deterioration In Acute Stroke Exploratory Analysis Of The Fast-Mag Randomized Clinical Trial |
|  | 2018 | Effect Of Alteplase Vs Aspirin On Functional Outcome For Patients With Acute Ischemic Stroke And Minor Nondisabling Neurologic Deficits The Prisms Randomized Clinical Trial |
|  | 2018 | Thrombectomy 6 To 24 Hours After Stroke With A Mismatch Between Deficit And Infarct |
|  | 2017 | Long-Term Outcomes Of Patent Foramen Ovale Closure Or Medical Therapy After Stroke |
|  | 2017 | Combined Intravenous Thrombolysis And Thrombectomy Vs Thrombectomy Alone For Acute Ischemic Stroke A Pooled Analysis Of The Swift And Star Studies |
|  | 2015 | Stent-Retriever Thrombectomy After Intravenous T-Pa Vs. T-Pa Alone In Stroke |
|  | 2015 | Prehospital Use Of Magnesium Sulfate As Neuroprotection In Acute Stroke |
|  | 2013 | Closure Of Patent Foramen Ovale Versus Medical Therapy After Cryptogenic Stroke |
|  | 2013 | A Trial Of Imaging Selection And Endovascular Treatment For Ischemic Stroke |
|  | 2012 | Solitaire Flow Restoration Device Versus The Merci Retriever In Patients With Acute Ischaemic Stroke (Swift): A Randomised, Parallel-Group, Non-Inferiority Trial |
|  | 1995 | Endarterectomy For Asymptomatic Carotid-Artery Stenosis |
| Berkhemer OA | 2022 | Prehospital Transdermal Glyceryl Trinitrate In Patients With Presumed Acute Stroke (Mr Asap): An Ambulance-Based, Multicentre, Randomised, Open-Label, Blinded Endpoint, Phase 3 Trial |
|  | 2021 | A Randomized Trial Of Intravenous Alteplase Before Endovascular Treatment For Stroke |
|  | 2018 | Association Of Reperfusion With Brain Edema In Patients With Acute Ischemic Stroke A Secondary Analysis Of The Mr Clean Trial |
|  | 2017 | Selection Of Patients For Intra-Arterial Treatment For Acute Ischaemic Stroke: Development And Validation Of A Clinical Decision Tool In Two Randomised Trials |
|  | 2017 | Two-Year Outcome After Endovascular Treatment For Acute Ischemic Stroke |
|  | 2016 | Effect Of Baseline Alberta Stroke Program Early Ct Score On Safety And Efficacy Of Intra-Arterial Treatment: A Subgroup Analysis Of A Randomised Phase 3 Trial (Mr Clean) |
|  | 2016 | Time To Reperfusion And Treatment Effect For Acute Ischemic Stroke A Randomized Clinical Trial |
|  | 2015 | A Randomized Trial Of Intraarterial Treatment For Acute Ischemic Stroke |
| Campbell BCV | 2022 | Endovascular Thrombectomy Versus Standard Bridging Thrombolytic With Endovascular Thrombectomy Within 4.5 H Of Stroke Onset: An Open-Label, Blinded-Endpoint, Randomised Non-Inferiority Trial |
|  | 2022 | Comparison Of Tenecteplase With Alteplase For The Early Treatment Of Ischaemic Stroke In The Melbourne Mobile Stroke Unit (Taste-A): A Phase 2, Randomised, Open-Label Trial |
|  | 2020 | Effect Of Intravenous Tenecteplase Dose On Cerebral Reperfusion Before Thrombectomy In Patients With Large Vessel Occlusion Ischemic Stroke The Extend-Ia Tnk Part 2 Randomized Clinical Trial |
|  | 2020 | Efficacy And Safety Of Nerinetide For The Treatment Of Acute Ischaemic Stroke (Escape-Na1): A Multicentre, Double-Blind, Randomised Controlled Trial |
|  | 2019 | Thrombolysis Guided By Perfusion Imaging Up To 9 Hours After Onset Of Stroke |
|  | 2019 | Mediation Of The Relationship Between Endovascular Therapy And Functional Outcome By Follow-Up Infarct Volume In Patients With Acute Ischemic Stroke |
|  | 2018 | Tenecteplase Versus Alteplase Before Thrombectomy For Ischemic Stroke |
|  | 2015 | Endovascular Therapy For Ischemic Stroke With Perfusion-Imaging Selection |
| Zaidat OO | 2019 | Association Of Thrombectomy With Stroke Outcomes Among Patient Subgroups Secondary Analyses Of The Defuse 3 Randomized Clinical Trial |
|  | 2015 | Effect Of A Balloon-Expandable Intracranial Stent Vs Medical Therapy On Risk Of Stroke In Patients With Symptomatic Intracranial Stenosis The Vissit Randomized Clinical Trial |
|  | 2014 | Aggressive Medical Treatment With Or Without Stenting In High-Risk Patients With Intracranial Artery Stenosis (Sammpris): The Final Results Of A Randomised Trial |
|  | 2013 | Endovascular Therapy After Intravenous T-Pa Versus T-Pa Alone For Stroke |
|  | 2012 | Solitaire Flow Restoration Device Versus The Merci Retriever In Patients With Acute Ischaemic Stroke (Swift): A Randomised, Parallel-Group, Non-Inferiority Trial |
|  | 2011 | Stenting Versus Aggressive Medical Therapy For Intracranial Arterial Stenosis |
| Goyal M | 2022 | Intensive Blood Pressure Control After Endovascular Thrombectomy For Acute Ischaemic Stroke (Enchanted2/Mt): A Multicentre, Open-Label, Blinded-Endpoint, Randomised Controlled Trial |
|  | 2021 | Assessment Of Optimal Patient Selection For Endovascular Thrombectomy Beyond 6 Hours After Symptom Onset A Pooled Analysis Of The Aurora Database |
|  | 2020 | Endovascular Thrombectomy With Or Without Intravenous Alteplase In Acute Stroke |
|  | 2020 | Efficacy And Safety Of Nerinetide For The Treatment Of Acute Ischaemic Stroke (Escape-Na1): A Multicentre, Double-Blind, Randomised Controlled Trial |
|  | 2019 | Mediation Of The Relationship Between Endovascular Therapy And Functional Outcome By Follow-Up Infarct Volume In Patients With Acute Ischemic Stroke |
|  | 2015 | Thrombectomy Within 8 Hours After Symptom Onset In Ischemic Stroke |
|  | 2015 | Stent-Retriever Thrombectomy After Intravenous T-Pa Vs. T-Pa Alone In Stroke |
|  | 2015 | Randomized Assessment Of Rapid Endovascular Treatment Of Ischemic Stroke |
|  | 2014 | Time To Angiographic Reperfusion And Clinical Outcome After Acute Ischaemic Stroke: An Analysis Of Data From The Interventional Management Of Stroke (Ims Iii) Phase 3 Trial |
|  | 2013 | Endovascular Therapy After Intravenous T-Pa Versus T-Pa Alone For Stroke |
|  | 2012 | Safety And Efficacy Of Na-1 In Patients With Iatrogenic Stroke After Endovascular Aneurysm Repair (Enact): A Phase 2, Randomised, Double-Blind, Placebo-Controlled Trial |
|  | 2022 | Trial Of Thrombectomy 6 To 24 Hours After Stroke Due To Basilar-Artery Occlusion |
| Jovin TG | 2022 | Effect Of Direct Transportation To Thrombectomy-Capable Center Vs Local Stroke Center On Neurological Outcomes In Patients With Suspected Large-Vessel Occlusion Stroke In Nonurban Areas The Racecat Randomized Clinical Trial |
|  | 2021 | Assessment Of Optimal Patient Selection For Endovascular Thrombectomy Beyond 6 Hours After Symptom Onset A Pooled Analysis Of The Aurora Database |
|  | 2019 | Mediation Of The Relationship Between Endovascular Therapy And Functional Outcome By Follow-Up Infarct Volume In Patients With Acute Ischemic Stroke |
|  | 2018 | Thrombectomy 6 To 24 Hours After Stroke With A Mismatch Between Deficit And Infarct |
|  | 2017 | Monitored Anesthesia Care Vs Intubation For Vertebrobasilar Stroke Endovascular Therapy |
|  | 2017 | Safety And Efficacy Of Thrombectomy In Acute Ischaemic Stroke (Revascat): 1-Year Follow-Up Of A Randomised Open-Label Trial |
|  | 2015 | Thrombectomy Within 8 Hours After Symptom Onset In Ischemic Stroke |
|  | 2015 | Stent-Retriever Thrombectomy After Intravenous T-Pa Vs. T-Pa Alone In Stroke |
|  | 2015 | Randomized Assessment Of Rapid Endovascular Treatment Of Ischemic Stroke |
|  | 2013 | Endovascular Therapy After Intravenous T-Pa Versus T-Pa Alone For Stroke |
|  | 2012 | Trevo Versus Merci Retrievers For Thrombectomy Revascularisation Of Large Vessel Occlusions In Acute Ischaemic Stroke (Trevo 2): A Randomised Trial |
|  | 2012 | Solitaire Flow Restoration Device Versus The Merci Retriever In Patients With Acute Ischaemic Stroke (Swift): A Randomised, Parallel-Group, Non-Inferiority Trial |
|  | 2005 | Comparison Of Warfarin And Aspirin For Symptomatic Intracranial Arterial Stenosis |
|  | 2022 | Intensive Blood Pressure Control After Endovascular Thrombectomy For Acute Ischaemic Stroke (Enchanted2/Mt): A Multicentre, Open-Label, Blinded-Endpoint, Randomised Controlled Trial |
| Wang YJ | 2022 | Time Course For Benefit And Risk With Ticagrelor And Aspirin In Individuals With Acute Ischemic Stroke Or Transient Ischemic Attack Who Carry Cyp2C19 Loss-Of-Function Alleles A Secondary Analysis Of The Chance-2 Randomized Clinical Trial |
|  | 2021 | Ticagrelor Versus Clopidogrel In Cyp2C19 Loss-Of-Function Carriers With Stroke Or Tia |
|  | 2021 | Efficacy And Safety Of Ticagrelor And Aspirin In Patients With Moderate Ischemic Stroke An Exploratory Analysis Of The Thales Randomized Clinical Trial |
|  | 2021 | Ticagrelor Added To Aspirin In Acute Ischemic Stroke Or Transient Ischemic Attack In Prevention Of Disabling Stroke A Randomized Clinical Trial |
|  | 2020 | Ticagrelor And Aspirin Or Aspirin Alone In Acute Ischemic Stroke Or Tia |
|  | 2020 | Endovascular Thrombectomy With Or Without Intravenous Alteplase In Acute Stroke |
|  | 2019 | Outcomes Associated With Clopidogrel-Aspirin Use In Minor Stroke Or Transient Ischemic Attack A Pooled Analysis Of Clopidogrel In High-Risk Patients With Acute Non-Disabling Cerebrovascular Events (Chance) And Platelet-Oriented Inhibition In New Tia And Minor Ischemic Stroke (Point) Trials |
|  | 2019 | Ticagrelor Plus Aspirin Versus Clopidogrel Plus Aspirin For Platelet Reactivity In Patients With Minor Stroke Or Transient Ischaemic Attack: Open Label, Blinded Endpoint, Randomised Controlled Phase Ii Trial |
|  | 2019 | Association Between Abcb1 Polymorphisms And Outcomes Of Clopidogrel Treatment In Patients With Minor Stroke Or Transient Ischemic Attack Secondary Analysis Of A Randomized Clinical Trial |
|  | 2018 | Effect Of A Multifaceted Quality Improvement Intervention On Hospital Personnel Adherence To Performance Measures In Patients With Acute Ischemic Stroke In China A Randomized Clinical Trial |
|  | 2018 | Dual Antiplatelet Therapy In Transient Ischemic Attack And Minor Stroke With Different Infarction Patterns Subgroup Analysis Of The Chance Randomized Clinical Trial |
|  | 2017 | Efficacy And Safety Of Ticagrelor Versus Aspirin In Acute Stroke Or Transient Ischaemic Attack Of Atherosclerotic Origin: A Subgroup Analysis Of Socrates, A Randomised, Double-Blind, Controlled Trial |
|  | 2016 | Ticagrelor Versus Aspirin In Acute Stroke Or Transient Ischemic Attack |
|  | 2016 | Association Between Cyp2C19 Loss-Of-Function Allele Status And Efficacy Of Clopidogrel For Risk Reduction Among Patients With Minor Stroke Or Transient Ischemic Attack |
|  | 2013 | Clopidogrel With Aspirin In Acute Minor Stroke Or Transient Ischemic Attack |
|  | 2011 | Extracranial-Intracranial Bypass Surgery For Stroke Prevention In Hemodynamic Cerebral Ischemia The Carotid Occlusion Surgery Study Randomized Trial |
| Powers WJ | 2011 | Extracranial-Intracranial Bypass Surgery For Stroke Prevention In Hemodynamic Cerebral Ischemia The Carotid Occlusion Surgery Study Randomized Trial |
| Johnston SC | 2022 | Infarct On Brain Imaging, Subsequent Ischemic Stroke, And Clopidogrel-Aspirin Efficacy A Post Hoc Analysis Of A Randomized Clinical Trial |
|  | 2021 | Ticagrelor Versus Clopidogrel In Cyp2C19 Loss-Of-Function Carriers With Stroke Or Tia |
|  | 2021 | Efficacy And Safety Of Ticagrelor And Aspirin In Patients With Moderate Ischemic Stroke An Exploratory Analysis Of The Thales Randomized Clinical Trial |
|  | 2021 | Ticagrelor Added To Aspirin In Acute Ischemic Stroke Or Transient Ischemic Attack In Prevention Of Disabling Stroke A Randomized Clinical Trial |
|  | 2020 | Ticagrelor And Aspirin Or Aspirin Alone In Acute Ischemic Stroke Or Tia |
|  | 2020 | Association Of Black Race With Early Recurrence After Minor Ischemic Stroke Or Transient Ischemic Attack Secondary Analysis Of The Point Randomized Clinical Trial |
|  | 2019 | Outcomes Associated With Clopidogrel-Aspirin Use In Minor Stroke Or Transient Ischemic Attack A Pooled Analysis Of Clopidogrel In High-Risk Patients With Acute Non-Disabling Cerebrovascular Events (Chance) And Platelet-Oriented Inhibition In New Tia And Minor Ischemic Stroke (Point) Trials |
|  | 2019 | Risk For Major Hemorrhages In Patients Receiving Clopidogrel And Aspirin Compared With Aspirin Alone After Transient Ischemic Attack Or Minor Ischemic Stroke A Secondary Analysis Of The Point Randomized Clinical Trial |
|  | 2019 | Ticagrelor Plus Aspirin Versus Clopidogrel Plus Aspirin For Platelet Reactivity In Patients With Minor Stroke Or Transient Ischaemic Attack: Open Label, Blinded Endpoint, Randomised Controlled Phase Ii Trial |
|  | 2019 | Association Between Abcb1 Polymorphisms And Outcomes Of Clopidogrel Treatment In Patients With Minor Stroke Or Transient Ischemic Attack Secondary Analysis Of A Randomized Clinical Trial |
|  | 2018 | Clopidogrel And Aspirin In Acute Ischemic Stroke And High-Risk Tia |
|  | 2017 | Efficacy And Safety Of Ticagrelor Versus Aspirin In Acute Stroke Or Transient Ischaemic Attack Of Atherosclerotic Origin: A Subgroup Analysis Of Socrates, A Randomised, Double-Blind, Controlled Trial |
|  | 2017 | Safety And Efficacy Of Natalizumab In Patients With Acute Ischaemic Stroke (Action): A Randomised, Placebo-Controlled, Double-Blind Phase 2 Trial |
|  | 2016 | Ticagrelor Versus Aspirin In Acute Stroke Or Transient Ischemic Attack |
|  | 2016 | Association Between Cyp2C19 Loss-Of-Function Allele Status And Efficacy Of Clopidogrel For Risk Reduction Among Patients With Minor Stroke Or Transient Ischemic Attack |
|  | 2013 | Clopidogrel With Aspirin In Acute Minor Stroke Or Transient Ischemic Attack |
|  | 2006 | Clopidogrel And Aspirin Versus Aspirin Alone For The Prevention Of Atherothrombotic Events |
